# The opportunities and challenges of social media in interstitial lung disease: a viewpoint

**DOI:** 10.1186/s12931-021-01843-4

**Published:** 2021-09-17

**Authors:** Japnam S. Grewal, Leticia Kawano-Dourado, Christopher J. Ryerson

**Affiliations:** 1 Department of Medicine, University of British Columbia, Vancouver, Canada; 2 HCor Research Institute, Hospital Do Coracao, Rua Abilio Soares, 250, 12º andar, São Paulo, SP 04005-909 Brazil; 3 Pulmonary Division, University of Sao Paulo, Sao Paulo, Brazil; 4 INSERM U1152, University of Paris, Paris, France; 5 Centre for Heart Lung Innovation, St. Paul’s Hospital, Vancouver, Canada

**Keywords:** Interstitial lung disease, Pulmonary fibrosis, Social media, Medical education, Health policy

## Abstract

Social media is an increasingly popular source of health information, and the rarity and complexity of interstitial lung disease (ILD) may particularly draw patients with ILD to social media for information and support. The objective of this viewpoint is to provide an overview of social media, explore the benefits and limitations of ILD-related social media use, and discuss future development of healthcare information on social media. We describe the value of integrating social media into the practice of ILD health professionals, including its role in information dissemination, patient engagement, knowledge generation, and formation of health policy. We also describe major challenges to expanded social media use in ILD, including limited access for some individuals and populations, abundance of misinformation, and concerns about patient privacy. Finally, for healthcare professionals looking to join social media, we provide practical guidance and considerations to optimize the potential benefits and minimize the potential pitfalls of social media.

**Supplementary Information:**

The online version contains supplementary material available at 10.1186/s12931-021-01843-4.

## Background

The recent rise of several social media platforms has transformed the Internet into an interactive network in which information is shared in multiple directions, setting the stage for creation and consumption of information on a large scale. The appeal of social media to individual users has rapidly established these platforms as major sources of knowledge acquisition, dissemination of information, networking, and advocacy. In the healthcare sector, healthcare professionals, patients, and others are constantly interacting in this online environment. Social media has in particular been transformational for patients with rare diseases, such as those with interstitial lung disease (ILD), allowing these patients to gather together to advocate, increase disease awareness, and expand or update their own knowledge on their disease. Concurrently, there are major benefits of social media for healthcare professionals, including advancing their own education, education of patients, and professional networking.

In this viewpoint, we provide an overview of social media in healthcare, and describe the benefits of ILD-specific social media use by healthcare professionals, including information dissemination, patient engagement, knowledge generation, and formation of health policy. We also discuss the limited access to social media for certain populations, abundance of misinformation, and concerns about patient privacy as major challenges of expanded social media use in ILD. We also offer guidance for new users, and present future directions in this area.

## Overview of social media

**Fig. 1:**
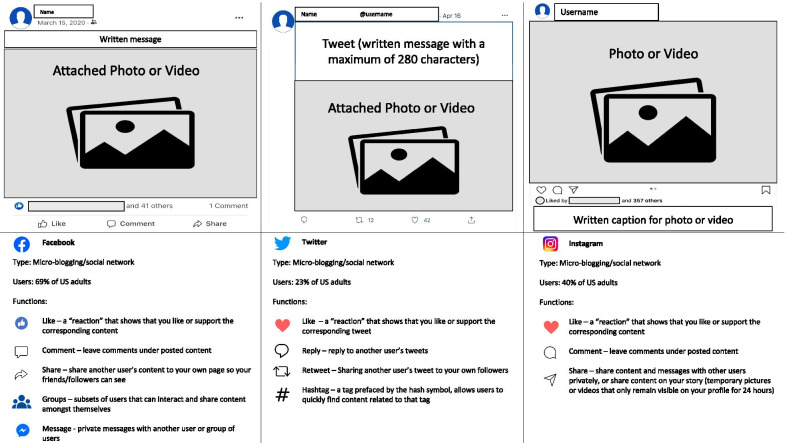
Major social media microblogging platforms. The top row represents the typical user interface for posted content on each platform. The bottom row depicts the key functions of the corresponding platform

Based on the Merriam-Webster Dictionary, social media is defined as “forms of electronic communication (such as websites for social networking and microblogging) through which users create online communities to share information, ideas, personal messages, and other content (such as videos)” [1]. Today, this occurs in many different social media platforms, each with unique functions and content. Facebook, Twitter, and Instagram are the most popular social networking and microblogging sites [2], as described in Fig. 1. LinkedIn was among the earliest mainstream social media platforms and remains popular as a networking site for professionals, job seekers, and recruiters. There are also video-based social media platforms, like YouTube, among the most visited websites on the Internet [2], and TikTok, an emerging social networking app that is based on creating short videos, often with dancing and trending soundtracks. Clubhouse has recently risen in popularity as a new invite-only audio-based social media app that hosts drop-in chats.

Social media use among the general population is increasing, with this trend not limited to youth and young adults. Compared to just 5% uptake in 2005, 72% of US adults now use some form of social media in 2021, including 73% of adults between the ages of 50–65 and 45% of those above the age of 65 [2], correlating with increasing internet and cellphone use during this time [3]. It is likely that the integration of social media into our lives will continue to increase, especially with younger generations coming of age with widespread availability of the Internet.

Beyond its ‘social’ impact, social media has significantly altered the way in which people consume healthcare information. The capabilities of social media platforms allow for a bidirectional flow of information between the “creators” and “consumers”, moving beyond the unidirectional flow of information characteristic of previous methods of information sharing.

This has transformed the patient-provider relationship, particularly when it comes to dissemination of health information. However, the open and relatively unregulated environment on social media has created issues for the general public, and there are also concerns that are specific to the healthcare sector or that are at least magnified in the healthcare setting. Below, we outline both the benefits and challenges of social media in the specific context of ILD.

## Benefits of social media in interstitial lung disease

The capabilities of social media are particularly useful for ILD patients and professionals given the rarity, complexity, and rapid evolution in our understanding of the multiple disorders that fall under the umbrella of ILD. Social media is a valuable tool for ILD information dissemination and acquisition, but also has additional benefits over traditional methods of health information sharing, such as facilitating patient engagement, fostering knowledge generation and research collaborations, and allowing for influence on health policy.

### Dissemination of information

A major convenience of social media for patients is the minimal time and effort that are required to access health information. This is of particular benefit to patients with ILD given the frequent centralization of services in tertiary centers and the challenges in attending in-person educational sessions or acquiring educational materials [4, 5]. Social media also offers the possibility for patients to follow ILD-specific users or groups that provide curated information relevant to a patient’s specific interest (Table 1). Previously shared information is typically stored in easily accessible and searchable archives specific to each account, thus supporting patients who wish to learn more about certain topics or discussions.

**Table 1:** Examples of ILD organizations and groups with social media content for patients

Organization/group	Social media platforms	Description
*ILD specific organizations / groups*		
Action for Pulmonary Fibrosis	Twitter (@ActionPFcharity)	Patient-led charity that provides education, support, and research funding
Breathe support	Facebook	Online network and support groups created by ILD patients and caregivers
Canadian Pulmonary Fibrosis Foundation	Facebook, Twitter (@THE_CPFF)	Canadian non-profit organization that provides education, support, and research funding
European IPF and Related Disorders Federation	Facebook, Twitter (@EU_IPFF), LinkedIn	Non-profit organization that unites 21 national ILD patient associations across Europe
PF Warriors	Facebook	International patient-led support group for ILD patients and caregivers
Pulmonary Fibrosis Foundation	Facebook, Twitter (@pfforg), Instagram (@pfforg), YouTube	American non-profit organization that provides education, support, and research funding
Pulmonary Fibrosis News	Twitter (@pulmonaryfibros), Instagram (@pulmonaryfibrosisnews)	Media company focused on reporting ILD-related news and research
*General respiratory organizations / groups*		
American Lung Association	Facebook, Twitter (@LungAssociation), Instagram (@lungassociation), YouTube, LinkedIn, TikTok (@americanlungassociation)	American non-profit organization providing respiratory-related education, support, advocacy, and research funding
British Lung Foundation	Facebook, Twitter (@lunguk), Instagram (@britishlungfoundation), YouTube	UK-based charity providing respiratory-related education, support, advocacy, and research funding
European Lung Foundation	Facebook, Twitter (@EuropeanLung), Instagram (@european_lung), YouTube, LinkedIn	European patient-led non-profit organization which provides respiratory-related education, support, advocacy, and research funding

### Patient engagement

Social media supports ILD patient engagement, defined as the active seeking out of disease-related information and involvement in healthcare decision-making, which results in better health outcomes and lower costs to the healthcare system [6–8]. When patients use social media for health-related purposes, they choose which users they follow, react to shared information, and share content that they found informative. If chosen, the anonymous nature of social media may make it more comfortable for patients to join conversations and speak up about questions or concerns they may have. Patients can also take on a leadership role by creating their own ILD-related social media content, or form connections that would otherwise be difficult to establish in-person given the relative rarity of ILD.

For healthcare professionals, social media creates a convenient forum that supports communication with a large and diverse audience. The community of ILD healthcare professionals continues to engage with patients, families, and others through various web forums and blogs that provide disease-related information and personal narratives as a method of supporting patients [9]. Such forums are typically hosted on micro-blogging social media sites such as Facebook, with added functions such as direct messaging or the ability to form “groups” that can improve patient comfort and dialogue compared to traditional websites or blogs.

Social media can also be used to support multiple components of patient care, including supplementation of in-person pulmonary rehabilitation and patient support groups. The benefits of pulmonary rehabilitation diminish after 12–18 months [10], while maintenance programs can extend this benefit [11]. Social media is well-suited to provide mechanisms through which a large number of patients can be supported in their ongoing pulmonary rehabilitation maintenance program to further extend this benefit. The applicability of social media to patient support groups is even more readily apparent [12–14], particularly for patients who struggle to attend in-person sessions and especially during the COVID-19 pandemic [15].

Social media can also be used to share information about clinical trials and other research opportunities for interested patients. This is specifically helpful given the rarity of some ILDs that often compromises study enrolment.

### Knowledge generation

Social media may also support research by facilitating survey-based data collection or big data analytics, and is of particular value in more rare diseases such as ILD given the difficulties with obtaining sufficient responses using traditional methods [16–19]. Surveys can be created using an established survey tool with a link distributed broadly on social media [16–18], and many social media sites also support creation of surveys on their own platforms. In a similar fashion, group or individual feedback can be easily solicited through social media, making it a potentially viable quality improvement tool.

The open bidirectional flow of information on social media also creates a stimulating environment for expert discussion on ILD-related topics. Compared to other methods of expert discussion, social media provides rapid and easy access to opinions from a large and diverse group of health professionals on contemporary controversies. This has been furthered by the COVID-19 pandemic, for example with large physician Facebook groups that were able to synthesize and disseminate expert opinion on various COVID-related clinical questions before peer-reviewed journals were able to do so [20]. Although these groups lack some advantages of the formal peer-review process, the rapid input from expert clinicians provided valuable discussion at a time when clinical experience frequently outpaced publication of evidence. Similar discourse between experts has also taken place on Twitter, and has served many other functions, including advocacy, health policy discussion, and peer support. The greater familiarity of social media ushered in by the COVID-19 pandemic will likely prompt a further increase in its use for other diseases such as ILD.

### Research collaborations and career development

The ease of communication and broad international presence on social media creates an environment that promotes collaborations among researchers. This is particularly true on Twitter, which has had significant uptake by the ILD community. While networking was previously centred around direct working relationships or in-person events such as conferences, social media removes geographical and time barriers to networking, creating opportunities for much larger and more diverse professional networks than was previously possible. These frequent and broad social media interactions centred around shared research interests likely increase the chances of forming fruitful research collaborations.

The structure of social media also breaks down the traditional hierarchy of academia by allowing trainees and early-career investigators to interact directly with more established key opinion leaders (KOLs). A large proportion of the KOLs in ILD are on Twitter. It is therefore possible for early-career health professionals to increase their visibility among established ILD KOLs through effective Twitter commentary and interaction, presenting a unique networking opportunity. This can in turn stimulate opportunities for career development, including mentorship, research collaborations, and leadership positions.

### Influencing health policy

Political discourse has largely migrated from being in print to being online, and particularly to social media platforms over the last several years. Different stakeholders increasingly use social media to present their views on major healthcare-related issues, including patients, healthcare professionals, political figures, “think tanks”, and media companies. Healthcare professionals are similarly able to bring public awareness to issues regarding healthcare access and provision, with a much greater opportunity to create change given the simultaneous presence of important stakeholders within their audience. This can include specific dialogue on individual diseases as well as more general societal issues.

## Challenges of social media in interstitial lung disease

The interactive bidirectional nature of social media that supports the aforementioned benefits is also the driver behind its main challenges. The online format and open environment have created issues for the general public, but there are also concerns that are specific to healthcare, including lack of access, misinformation, and compromise of patient privacy.

### Lack of access or availability of social media content

Limited access to social media can occur due to a lack of internet or a necessary device. Geographic limitations to internet access are an additional challenge in many rural communities, again excluding a specific subset of patients that is likely those in most need. Third, age is a major driver in access to internet and a capable device. Even though social media usage is increasing in the elderly, 27% of Americans above the age of 65 years continue to lack internet access and 47% do not have a smartphone, consistent with 55% of those above 65 years of age not using social media [2, 3]. This is of particular importance in ILD, as many ILDs disproportionately affect older people [21, 22]. Similarly, lack of tech savviness or experience with social media reduces the level of participation, and is again more likely to affect older people who are most frequently affected by ILD [21, 22].

Even if the means to access social media are obtained, the availability of ILD-specific social media content remains an issue [23, 24]. The limited ILD content that is available tends to be focused on idiopathic pulmonary fibrosis (IPF), without disease-specific information on other ILDs. Language is also a significant barrier as most online resources on ILDs are only available in English. The lack of targeted health information on social media for non-IPF and non-English speaking ILD patients may lead them to seek out information from non-trusted sources, perpetuating the spread of misinformation.

### Misinformation

The paucity of trusted and high-quality online information is in part related to the non-curated nature of social media, failure of publishers to prioritize social media content as an information source, and the resultant lack of updates to their content as our understanding of ILD evolves [23–25]. Social media creates an environment where the most popular or shared posts can dominate the discussion, even if these are not scientifically accurate, contributing to the preponderance of attention-grabbing “click-bait” headlines that promote alarming and often unfounded concerns on health-related topics [26]. Given the abundance of opinions provided online, patients also have the opportunity to seek out “echo chambers” that simply perpetuate their own beliefs, which again may not be scientifically valid. Instant access to false claims and advertising of non-recommended therapies on social media poses a significant risk to public health, and may be accentuated in rare diseases such as ILD. Social media allows this misinformation to be amplified and broadcast rapidly to large audiences, further increasing the risk it poses.

### Patient privacy and confidentiality

Users typically relinquish some personal privacy when attempting to take full advantage of social media. For example, ILD-related social media use by patients may publicly identify them as having ILD, which can have significant psychological implications and may create a disease stigma [27, 28]. This public information can further impact relationships, ability to secure healthcare or life insurance, or success in the workplace. Another unintended effect of using social media for health purposes is that groups with ulterior motives may initiate unwanted interactions with patients to advance their own agendas, representing the twenty-first century version of the snake oil salesperson. There is also a potential lack of safety when interacting on social media, with the potential for random attacks from those who have differing opinions as well as hacks or cyberattacks that can lead to financial losses or exposure of sensitive information. Public concerns with data privacy have significantly increased in the last years, generating legal discussions, which may create a safer online environment for the individual user.

## Future directions

**Fig. 2:**
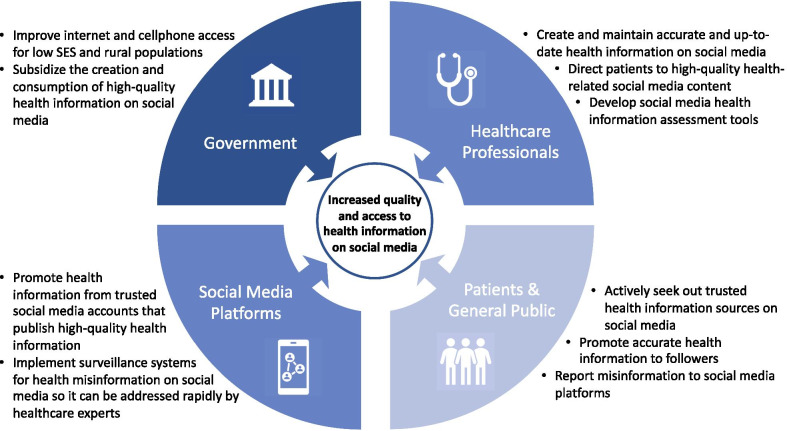
Action items for stakeholders of healthcare-related social media use. *SES* = socioeconomic status

Applications of social media in healthcare will continue to evolve given the constant updating of established social media platforms and rapid rise of new ones such as TikTok. Social media use for healthcare purposes will also increase as younger generations become more frequent users of the healthcare system. These trends highlight the importance of optimizing these platforms for this purpose, including the need to reduce misinformation and increase access for marginalized populations. To achieve these objectives, engagement from multiple stakeholders is needed, including government officials, social media platforms, healthcare professionals, and the general public (Fig. 2).

### Quality control on social media

Given the abundance of health-related information on existing social media platforms, efficient and precise quality assessment tools are need to objectively assess the quality of health information released on social media. With these tools, users would be better equipped to identify false information and poor-quality sources, which can then be used by social media platforms to minimize the public exposure to such accounts while conversely promoting high-quality sources. Social media platforms should also actively work with the healthcare community to implement surveillance systems where health misinformation can be rapidly identified and addressed before it goes ‘viral’ and impacts a large audience on social media [29]. This active approach to curbing misinformation raises important questions such as whether social media platforms should have the authority to ‘silence’ accounts that consistently produce misinformation, and the criteria they would use to do so.

### Increasing access to accurate health information for marginalized populations

Continued efforts are needed to increase internet and cellphone access in communities and populations with lower socioeconomic status in an attempt to improve patient health and safety [30–32]. Realizing such goals would help ensure that these groups are active participants in the new health information paradigm on social media. Other potential strategies should be considered to help mitigate financial challenges with accessing the internet and social media platforms in particular, including subsidizing internet or cellphone data usage for healthcare or health information purposes, and ensuring healthcare content on social media is compatible with cheaper and older devices. Non-financial barriers must also be considered, such as the variable cultural and educational background of ILD patients. For example, providing ILD content in multiple languages and at an appropriate reading level can broaden the impact and uptake of ILD health information.

## Joining social media as a healthcare professional

Many healthcare professionals choose to join social media for the first time given the benefits discussed above, while others may transition their existing personal accounts to a more professional orientation. For both groups, there are key considerations that will maximize the impact and outreach of one’s social media content, while also minimizing the potential for encountering some of the challenges that social media can create.

### Things to consider before joining social media

The goals of one’s social media use should be identified prior to creating a social media account. For example, is the use of social media primarily for a professional purpose, or purely for personal life? Is the intended objective best suited for social media, or would other tools or methods be more appropriate? Answering these types of questions will help determine if embarking on social media is worthwhile for the identified purpose and will also identify which platform is best suited for this use. For instance, YouTube is the most suitable option for creating and posting patient information videos, Twitter may be best for networking with other researchers, and LinkedIn is preferable for job seeking.

In addition to the many potential positives, social media is a powerful spotlight that can work against the user depending on the content and interactions they choose to create. This increased exposure and potential for a permanent record of any negative interactions necessitates careful reflection by healthcare professionals prior to sharing content on social media. Posted content can be hard to erase from the Internet even if it is deleted from the author’s account, and there have been numerous examples of professionals jeopardizing their careers and losing their jobs based on ill-advised social media posts. Many of these examples stem from ignorance concerning a specific topic or language, rather than intentionally harmful statements, and it is thus critical for users to carefully consider how their opinions and statements could be perceived from a diverse audience that has a multitude of different perspectives.

Similarly, the legal implications of social media use for the purposes of healthcare should be considered. Healthcare professionals often find themselves as the recipient of requests for medical advice from strangers on social media. In general, healthcare professionals should refrain from providing medical advice on social media given the possible liabilities, and patients should instead be re-directed to their own healthcare providers for personal healthcare advice. Depending on the jurisdiction, it may also be advisable for healthcare professionals to indicate that the content on their personal account does not reflect the views of their employers, affectively creating legal separation. This is of particular importance for users who express personal views or content beyond healthcare on their social media accounts.

### Getting started on social media

After making the decision to join social media, identifying the intended goals, and selecting a social media platform, healthcare professionals should proceed with caution through the account setup process in order to ensure the user’s best foot is put forward with their initial profile. Major components of profiles on most social media platforms include a username, biography, profile picture and cover photo (Additional file 1; Supplementary Appendix: Figure S1). Further details on considerations when setting up a social media account can be found in the Supplementary Appendix. Following the creation of an account, users should familiarize themselves with the functions and typical format of discourse on the selected social media platform. Each social media platform has their own particularities, but there are several universal suggestions for healthcare professionals starting on social media (Table 2).

**Table 2:** Tips for healthcare professionals starting on social media

Follow accounts that align with your research, clinical, and personal interests. This includes scientific journals, societies, key opinion leaders, etc
Before creating your own content, review the content posted by accounts with similar objectives as yours and how these users interact with their audience
Politely engage in conversations on others’ posts by replying or sharing comments on the topic being discussed: this is a way to start to be seen on social media
Re-tweet and share content from others in order to show you liked the content and to provide greater visibility of the original account to your colleagues
Do not contribute to the chain of misinformation. Avoid quick re-posting/re-tweeting and make sure you only post/tweet content you have read and fully understand
Familiarize yourself with reporting and blocking features in order to remove harmful comments and ‘spam’ from your social media feed

## Conclusions

In summary, social media has emerged as a significant space for health information sharing for both patients and healthcare professionals. Healthcare professionals focused on ILD can use social media to disseminate disease information, engage patients, generate new knowledge, and influence health policy. Nonetheless, lack of access, misinformation, and patient privacy remain significant challenges when integrating social media into patient care. Enhanced assessment of healthcare information sources on social media is needed to address misinformation, and active participation by stakeholders at multiple levels is required to increase access to health information on social media. Given the dynamic crowdsourced nature of social media, it is essential that the ILD community collectively strives to ensure a safe and productive online environment that serves the needs of patients, professionals, and other stakeholders.

## Supplementary Information

**Additional file 1. Supplementary Appendix: Figure S1.** Major components of a Twitter account profile. The components are marked by overlaid red text. A verified badge is given to accounts of public interest that have had their authenticity verified by Twitter.

## Data Availability

Not applicable.

## Abbreviations

ILD: Interstitial lung disease
IPF: Idiopathic pulmonary fibrosis
KOLs: Key opinion leaders

## References

[1] Merriam-Webster.com. Social media. http://www.merriamwebster.com/dictionary/socialmedia. Accessed 5 Apr 2021.

[2] Auxier B, Anderson M. Social media use in 2021. Pew Research Center. 2021. https://www.pewresearch.org/internet/2021/04/07/social-media-use-in-2021. Accessed 5 Apr 2021.

[3] Anderson M. Mobile technology and home broadband 2019. Pew Research Center. 2019. https://www.pewresearch.org/internet/2019/06/13/mobile-technology-and-home-broadband-2019. Accessed 5 Apr 2021.

[4] Johannson KA, Lethebe BC, Assayag D, Fisher JH, Kolb M, Morisset J, Shapera S, Gershon AS, Hambly N, Khalil N, To T, Fell CD, Cox G, Manganas H, Halayko AJ, Marcoux V, Sadatsafavi M, Wilcox PG, Bertazzon S, Ryerson CJ. Travel distance to subspecialty clinic and outcomes in patients with fibrotic interstitial lung disease. Ann Am Thorac Soc. 2021. 10.1513/AnnalsATS.202102-216OC 34033739

[5] Grewal JS, Morisset J, Fisher JH, Churg AM, Bilawich AM, Ellis J, English JC, Hague CJ, Khalil N, Leipsic J, Mayo J, Muller NL, Murphy D, Wright JL, Ryerson CJ (2019). Role of a regional multidisciplinary conference in the diagnosis of interstitial lung disease. Ann Am Thorac Soc.

[6] Osborn R, Squires D (2012). International perspectives on patient engagement: results from the 2011 Commonwealth Fund survey. J Ambul Care Manag.

[7] Coulter A, Ellins J (2007). Effectiveness of strategies for informing, educating, and involving patients. BMJ.

[8] Coulter A (2012). Patient engagement—what works?. J Ambul Care Manag.

[9] Albright K, Walker T, Baird S, Eres L, Farnsworth T, Fier K, Kervitsky D, Korn M, Lederer DJ, McCormick M, Steiner JF, Vierzba T, Wamboldt FS, Swigris JJ (2016). Seeking and sharing: why the pulmonary fibrosis community engages the web 2.0 environment. BMC Pulm Med.

[10] Spruit MA, Singh SJ, Garvey C, ZuWallack R, Nici L, Rochester C, Hill K, Holland AE, Lareau SC, Man WD, Pitta F, Sewell L, Raskin J, Bourbeau J, Crouch R, Franssen FM, Casaburi R, Vercoulen JH, Vogiatzis I, Gosselink R, Clini EM, Effing TW, Maltais F, van der Palen J, Troosters T, Janssen DJ, Collins E, Garcia-Aymerich J, Brooks D, Fahy BF, Puhan MA, Hoogendoorn M, Garrod R, Schols AM, Carlin B, Benzo R, Meek P, Morgan M, Rutten-van Mölken MP, Ries AL, Make B, Goldstein RS, Dowson CA, Brozek JL, Donner CF, Wouters EF, ATS/ERS Task Force on Pulmonary Rehabilitation (2013). An official American Thoracic Society/European Respiratory Society statement: key concepts and advances in pulmonary rehabilitation. Am J Respir Crit Care Med.

[11] Güell MR, Cejudo P, Ortega F, Puy MC, Rodríguez-Trigo G, Pijoan JI, Martinez-Indart L, Gorostiza A, Bdeir K, Celli B, Galdiz JB (2017). Benefits of long-term pulmonary rehabilitation maintenance program in patients with severe chronic obstructive pulmonary disease. Three-year follow-up. Am J Respir Crit Care Med.

[12] Athanasiadis DI, Roper A, Hilgendorf W, Voss A, Zike T, Embry M, Banerjee A, Selzer D, Stefanidis D. Facebook groups provide effective social support to patients after bariatric surgery. Surg Endosc. 2020. 10.1007/s00464-020-07884-y 32780242

[13] Apperson A, Stellefson M, Paige SR, Chaney BH, Chaney JD, Wang MQ, Mohan A (2019). Facebook groups on chronic obstructive pulmonary disease: social media content analysis. Int J Environ Res Public Health.

[14] Muhammad S, Allan M, Ali F, Bonacina M, Adams M (2014). The renal patient support group: supporting patients with chronic kidney disease through social media. J Ren Care.

[15] Wong AW, Fidler L, Marcoux V, Johannson KA, Assayag D, Fisher JH, Hambly N, Kolb M, Morisset J, Shapera S, Ryerson CJ (2020). Practical considerations for the diagnosis and treatment of fibrotic interstitial lung disease during the coronavirus disease 2019 pandemic. Chest.

[16] Schumacher KR, Stringer KA, Donohue JE, Yu S, Shaver A, Caruthers RL, Zikmund-Fisher BJ, Fifer C, Goldberg C, Russell MW (2014). Social media methods for studying rare diseases. Pediatrics.

[17] Chatterjee S, Humby T, Davies W (2016). Behavioural and psychiatric phenotypes in men and boys with X-linked ichthyosis: evidence from a worldwide online survey. PLoS ONE.

[18] Ramo DE, Prochaska JJ (2012). Broad reach and targeted recruitment using Facebook for an online survey of young adult substance use. J Med Internet Res.

[19] Davies W (2016). Insights into rare diseases from social media surveys. Orphanet J Rare Dis.

[20] Shekar S, Aravantagi A (2020). Are social media groups the novel physician lounges to combat COVID times?. J Gen Intern Med.

[21] Duchemann B, Annesi-Maesano I, Jacobe de Naurois C, Sanyal S, Brillet PY, Brauner M, Kambouchner M, Huynh S, Naccache JM, Borie R, Piquet J, Mekinian A, Virally J, Uzunhan Y, Cadranel J, Crestani B, Fain O, Lhote F, Dhote R, Saidenberg-Kermanac'h N, Rosental PA, Valeyre D, Nunes H (2017). Prevalence and incidence of interstitial lung diseases in a multi-ethnic county of Greater Paris. Eur Respir J.

[22] Raghu G, Remy-Jardin M, Myers JL, Richeldi L, Ryerson CJ, Lederer DJ, Behr J, Cottin V, Danoff SK, Morell F, Flaherty KR, Wells A, Martinez FJ, Azuma A, Bice TJ, Bouros D, Brown KK, Collard HR, Duggal A, Galvin L, Inoue Y, Jenkins RG, Johkoh T, Kazerooni EA, Kitaichi M, Knight SL, Mansour G, Nicholson AG, Pipavath SNJ, Buendía-Roldán I, Selman M, Travis WD, Walsh S, Wilson KC, American Thoracic Society, European Respiratory Society, Japanese Respiratory Society, and Latin American Thoracic Society (2018). Diagnosis of idiopathic pulmonary fibrosis an official ATS/ERS/JRS/ALAT clinical practice guideline. Am J Respir Crit Care Med.

[23] Fisher JH, O'Connor D, Flexman AM, Shapera S, Ryerson CJ (2016). Accuracy and reliability of internet resources for information on idiopathic pulmonary fibrosis. Am J Respir Crit Care Med.

[24] Grewal JS, Fisher JH, Ryerson CJ. An updated assessment of online information on idiopathic pulmonary fibrosis. Ann Am Thorac Soc. 2021. 10.1513/AnnalsATS.202012-1479RL 33567231

[25] Goobie GC, Guler SA, Johannson KA, Fisher JH, Ryerson CJ (2019). YouTube videos as a source of misinformation on idiopathic pulmonary fibrosis. Ann Am Thorac Soc.

[26] Wang Y, McKee M, Torbica A, Stuckler D (2019). Systematic literature review on the spread of health-related misinformation on social media. Soc Sci Med.

[27] Berger BE, Kapella MC, Larson JL (2011). The experience of stigma in chronic obstructive pulmonary disease. West J Nurs Res.

[28] Johnson JL, Campbell AC, Bowers M, Nichol AM (2007). Understanding the social consequences of chronic obstructive pulmonary disease: the effects of stigma and gender. Proc Am Thorac Soc.

[29] Scales D, Gorman J, Jamieson KH. The COVID-19 infodemic—applying the epidemiologic model to counter misinformation. N Engl J Med. 2021. 10.1056/NEJMp2103798 33979506

[30] Benda NC, Veinot TC, Sieck CJ, Ancker JS (2020). Broadband internet access is a social determinant of health!. Am J Public Health.

[31] Sieck CJ, Sheon A, Ancker JS, Castek J, Callahan B, Siefer A (2021). Digital inclusion as a social determinant of health. NPJ Digit Med.

[32] Early J, Hernandez A. Digital disenfranchisement and COVID-19: broadband internet access as a social determinant of health. Health Promot Pract. 2021. 10.1177/15248399211014490 33955266

