# Supplementary material for: The opportunities and challenges of social media in interstitial lung disease: a viewpoint
Source: Respir Res. 2021 Sep 17;22:247. doi: 10.1186/s12931-021-01843-4 (PMC8448389; doi:10.1186/s12931-021-01843-4)
Supplement: Supplementary file 1 — Additional file 1. Supplementary Appendix: Figure S1. Major components of a Twitter account profile. The components are marked by overlaid red text. A verified badge is given to accounts of public interest that have had their authenticity verified by Twitter. [file 12931_2021_1843_MOESM1_ESM.docx]

**The opportunities and challenges of social media in interstitial lung disease: a viewpoint**

Japnam S. Grewal, Leticia Kawano-Dourado, Christopher J. Ryerson

**-- Online Additional Data --**

**SETTING UP A SOCIAL MEDIA ACCOUNT AS A HEALTHCARE PROFESSIONAL**

After making the decision to join social media, identifying the intended goals, and selecting a social media platform, users should proceed with caution through the account setup process in order to ensure the user’s best foot is put forward with their initial profile [**Figure E1**]. Many social media platforms, including Twitter and Instagram, require the creation of a username that is separate from the user’s first and last name on the account. Apart from the character limits for usernames that are unique to each social media platform, almost any title can be chosen for the username; however, it is advisable to pick something that is short and easily identifiable that will support name recognition. This often means including at least part of one’s legal name in the username, and sometimes including credentials and/or areas of focus in the name or username associated with the account so the audience can identify the background of the user and easily validate their legitimacy as a healthcare professional.

A small biography or personal description is available in many social media platforms, with this brief description of the account requiring considerable thought. This is where users should provide details of who is managing the account and what content will be covered in posts. A personal touch, such as adding hobbies or passions, can also help the audience get a sense of the user’s life outside of medicine, which enhances interpersonal connection. A frequent dilemma encountered by healthcare professionals is how much of their personal lives to share on social media accounts that are professionally oriented. This balance is different for every individual, with the use of separate accounts or different social media platforms for professional and personal use being a frequent compromise. Most social media platforms have privacy settings that can be used to filter the audience that can view a user’s profile and the content they post. Any content posted when the account is ‘public’ is globally accessible, and thus the implications of this should be considered before posting personal information or content.

**Figure S1.** Major components of a Twitter account profile. The components are marked by overlaid red text. A verified badge is given to accounts of public interest that have had their authenticity verified by Twitter.
